# Diet quality and depressive symptoms in adolescence: no cross-sectional or prospective associations following adjustment for covariates

**DOI:** 10.1017/S1368980018001179

**Published:** 2018-05-16

**Authors:** Eleanor M Winpenny, Anne-Laura van Harmelen, Martin White, Esther MF van Sluijs, Ian M Goodyer

**Affiliations:** 1 MRC Epidemiology Unit and Centre for Diet and Activity Research, School of Clinical Medicine, University of Cambridge, Addenbrooke’s Hospital, Box 285, Hills Road, Cambridge CB2 0QQ, UK; 2 Developmental Psychiatry, Department of Psychiatry, University of Cambridge, Cambridge, UK

**Keywords:** Diet, Depression, Adolescence, Fish, Fruits, Vegetables, Mediterranean diet score, Prospective

## Abstract

**Objective:**

Adolescence is a critical period for development of depression and understanding of behavioural risk factors is needed to support appropriate preventive strategies. We examined associations between adolescent diet quality and depressive symptoms, cross-sectionally and prospectively, in a large community cohort, adjusting for behavioural and psychosocial covariates.

**Design:**

Prospective community-based cohort study (ROOTS).

**Setting:**

Secondary schools in Cambridgeshire and Suffolk, UK.

**Subjects:**

Study participants (*n* 603) who completed 4 d diet diaries at age 14 years and reported depressive symptoms (Moods and Feelings Questionnaire (MFQ)) at 14 and 17 years of age.

**Results:**

Diet data were processed to derive a Mediterranean diet score (MDS) and daily servings of fruit and vegetables, and fish. At age 14 years, a negative association between fruit and vegetable intake and MFQ score was seen in the unadjusted cross-sectional regression model (*β*=−0·40; 95 % CI −0·71,−0·10), but adjustment for behavioural covariates, including smoking and alcohol consumption, attenuated this association. Fish intake and MDS were not cross-sectionally associated with MFQ score. No prospective associations were found between MDS, fruit and vegetable intake or fish intake and later MFQ score.

**Conclusions:**

Diet quality was not associated with depressive symptoms in mid-adolescence. Previously reported associations in this age range may be due to confounding. Further longitudinal studies are needed that investigate associations between adolescent diet and depression across different time frames and populations, ensuring appropriate adjustment for covariates.

Adolescence is a critical period for development of depression, with estimated 1-year prevalence increasing from less than 1 % in childhood to 4–5 % by mid- to late adolescence^(^ ^1^ ^)^. Adolescent depression is a risk factor for depression recurrence later in life^(^ ^2^ ^)^, as well as for a wide range of other mental health disorders in adulthood, for example anxiety disorders, substance-related disorders and bipolar disorder^(^ ^1^ ^)^. As mental disorders are the number one global contributor to loss of disability-adjusted life years in those aged 15–49 years^(^ ^3^ ^)^, there is an urgent need to formulate appropriate preventive strategies. Childhood and adolescence are periods of crucial importance for understanding the development of depressive illness and the contribution of behavioural risk.

Diet quality is one behavioural risk factor that has been associated with depression. Reviews and meta-analyses of published evidence from cross-sectional and prospective studies in adults have concluded that a healthy diet in adulthood is associated with a reduced risk of depression^(^ ^4^ ^)^. In particular, adherence to a Mediterranean diet (characterized by higher intakes of fruits, vegetables, legumes, nuts, whole grains, fish and monounsaturated fats, and lower intakes of red and processed meats and alcohol)^(^ ^5^ ^)^, fruit and vegetable intake^(^ ^6^ ^)^ and fish intake^(^ ^7^ ^)^ have all been associated with reduced risk of depression in adults. Putative mechanisms for such associations include decreases in adiposity and inflammation. Increased adiposity, frequently associated with poor quality of diet, has been associated with increased risk of depression^(^ ^8^ ^–^ ^10^ ^)^. Such an association between adiposity and depression may be mediated by inflammation: adipose tissue is known to release inflammatory cytokines and certain inflammatory markers have been associated with increased risk of depression^(^ ^11^ ^,^ ^12^ ^)^. In addition, higher diet quality has been directly associated with lower concentrations of inflammatory markers after adjustment for adiposity^(^ ^13^ ^)^, allowing the possibility of a more direct pathway between diet quality and depression.

Studies of associations between diet and depression in adolescence are more limited. Two recent systematic reviews addressed the relationship between diet and depression in adolescents^(^ ^14^ ^,^ ^15^ ^)^, reporting support for an association between a healthy diet and lower levels of depression, but based on limited evidence. Across these reviews only three prospective studies were identified, two of which reported negative associations between diet quality and measures of mental health related to depression^(^ ^16^ ^–^ ^18^ ^)^. Each of these prospective studies had limitations in dietary assessment or covariate adjustment. Only one study^(^ ^17^ ^)^ used a comprehensive measure of dietary intake, which aimed to assess all foods and drinks consumed over a defined period (by an FFQ) and adjusted associations for total energy intake, while the remaining two studies assessed diet based on a limited number of questionnaire items. Additionally, the studies showed limitations in covariate adjustment, with only one study including a range of health behaviours (physical activity, smoking, drinking and drug use) and psychosocial factors (parental conflict and social support) as covariates^(^ ^18^ ^)^.

In the present study we analysed data on a cohort of adolescents (the ROOTS study^(^ ^19^ ^,^ ^20^ ^)^) to gain a better understanding of the associations between diet and depression in adolescence. The ROOTS study included a comprehensive dietary assessment at age 14 years, a validated measure of depressive symptoms at ages 14 and 17 years^(^ ^21^ ^,^ ^22^ ^)^, and included a wide range of additional measures allowing adjustment for sociodemographic, anthropometric, behavioural and psychological factors in longitudinal analyses. Based on associations seen in adults, we characterized diet quality using a Mediterranean diet score (MDS)^(^ ^5^ ^)^, as well as studying independent associations of intake of fruit and vegetables^(^ ^6^ ^)^ and intake of fish^(^ ^7^ ^)^ with depressive symptoms in this cohort. The MDS is well established and validated as a measure of overall diet quality in US and European populations^(^ ^23^ ^–^ ^25^ ^)^ and has been shown to perform comparably to other diet quality scores in US populations^(^ ^24^ ^)^. Our aim in the present study was to explore cross-sectional and prospective associations between diet and depressive symptoms, to address our main question: What is the association between diet quality (MDS, fruit and vegetables, fish) at age 14 years and development of depressive symptoms from 14 to 17 years of age?

## Methods

### Study population and design

The ROOTS study is a longitudinal cohort study of adolescent development, focusing on the risk patterns and processes for the emergence of psychopathology during adolescence^(^ ^19^ ^,^ ^20^ ^)^. Participants (*n* 1238) were recruited at age 14 years through secondary schools in Cambridgeshire and Suffolk, UK, from 2005 to 2007. At mean age 14·5 years (sd 3·5 months), participants completed self-reported demographic and psychosocial measures. Six months later, participants were invited to take part in a sub-study focusing on diet, physical activity and body composition, and 932 (75·3 %) participants completed a diet diary. These data collection periods are collectively referred to as ‘baseline’ in the present analyses. The cohort was followed for 3 years from study entry to mean age 17·5 years (sd 4·1 months).

### Dietary measurement and assessment of diet quality

Participants were asked to complete a 4 d diet diary, including two weekdays and two weekend days, reporting estimated portion sizes in terms of small, medium or large, household measures or as individual items. Training was provided, involving practice diary completion and feedback from the research team, and on return of completed diary and physical activity monitor, participants received a £30 voucher. Diets were coded at the Medical Research Council (MRC) Human Nutrition Research Unit (Cambridge, UK) using the Diet-In-Nutrients-Out (DINO) system^(^ ^26^ ^)^. Portion weights were approximated using published values for children^(^ ^27^ ^–^ ^29^ ^)^.

Participants with three or more days of diet diary data available were included in the analysis. Extreme misreporters, those reporting an energy intake of <2092 kJ/d (<500 kcal/d) or >14 644 kJ/d (>3500 kcal/d), were excluded^(^ ^30^ ^)^. Diet data were processed to give a measure of diet quality, known as the alternative Mediterranean diet score (hereafter referred to as MDS)^(^ ^13^ ^)^. Median and interquartile range of each of the variables making up the MDS were calculated and are reported to give an indication of the distribution of intakes. Median and interquartile range are reported, rather than mean and standard deviation, due to the skewed distribution of these variables. To create the MDS score, data were first adjusted to an energy intake of 7531 kJ/d (1800 kcal/d) using the residual method^(^ ^23^ ^,^ ^31^ ^)^ and individuals were then scored as described previously^(^ ^13^ ^)^, based on their intake of nine food items (vegetables, legumes, fruit, nuts, whole grains, red and processed meat, fish, ratio of monounsaturated to saturated fat, ethanol) in comparison with the population median. We included a minor adaptation for adolescents, following van de Laar *et al*.^(^ ^25^ ^)^, such that ethanol intake was scored as 1 point for no intake and 0 for any other level of intake. Our assessment of whole grains included wholegrain breakfast cereals and breads only, since information on all whole grains consumed was not available. Average daily intakes of fruit and vegetables, fish and total energy were calculated and data on fruit and vegetables and fish converted into daily servings, using a serving size of 80 g for fruit and vegetables and 140 g for fish^(^ ^32^ ^)^.

### Depressive symptoms

At baseline and follow-up, participants completed the Moods and Feelings Questionnaire (MFQ)^(^ ^33^ ^)^, a thirty-three-item self-report measure of depressive symptoms, including factors such as low mood, loss of appetite, anhedonia, irritability and restlessness. Against each item, participants reported their mood over the previous two weeks on a three-point scale (mostly/sometimes/never), giving an overall score ranging from 0 to 66. Higher summed MFQ scores indicate more depressive symptoms. The MFQ has moderate to high criterion validity as a screen for adolescents with unipolar depression, with an optimal cut-point of ≥20 on the MFQ suggested to discriminate participants with any mood disorder from those with no mood disorder and an optimal cut-point of ≥29 on the MFQ suggested to discriminate participants having current major depressive episodes^(^ ^21^ ^)^.

### Covariates

A wide range of covariates were included to adjust for confounding of associations by variables likely to be associated with both diet and depression, as well as to increase the precision of estimates by adjustment for covariates strongly associated with the outcome variable. Based on associations reported in published literature, covariates included sociodemographic factors (sex, socio-economic status (SES))^(^ ^1^ ^,^ ^34^ ^,^ ^35^ ^)^, behavioural factors (smoking level, level of alcohol consumption, physical activity, sleep)^(^ ^36^ ^–^ ^41^ ^)^, psychosocial factors (friendship quality, self-esteem, family functioning)^(^ ^42^ ^,^ ^43^ ^)^, anthropometric factors (percentage body fat)^(^ ^9^ ^,^ ^44^ ^)^, medication use and total energy intake^(^ ^45^ ^)^. Adjustment for total energy intake as a covariate was not included in associations between MDS and depressive symptoms, since adjustment for total energy was already included in the MDS.

Data on all covariates were collected at baseline. Participants self-reported their sex. Neighbourhood-level SES was assessed using the ACORN index to categorize UK postcodes into five categories^(^ ^46^ ^)^. These categories were further collapsed to give three categories: high (categories 1/2), middle (category 3) and low SES (categories 4/5).

Cigarette smoking (cigarettes smoked per day) and level of alcohol consumption (total number of days when alcohol was consumed, across four categories) were assessed by questionnaire. The level of alcohol consumption was adopted as a covariate in addition to inclusion of alcohol in the MDS, since alcohol in the MDS was only a binary variable assessing any consumption *v.* no consumption, whereas as a covariate we adjusted for the level of consumption. Physical activity was recorded using a combined heart rate and movement sensor (Actiheart, CamNtech Ltd, Papworth, UK) for four consecutive days, as reported previously^(^ ^47^ ^)^. Valid Actiheart monitor wear was defined as ≥48 h of data, including at least 8 h of data from all quadrants of a 24 h day (≥8 h from morning (03.00–09.00 hours), noon (09.00–15.00 hours), afternoon (15.00–21.00 hours) and night (21.00–03.00 hours) time periods)^(^ ^47^ ^)^. Moderate-to-vigorous physical activity was defined as time spent above 4 MET (metabolic equivalents of task). Sleep data combined data from self-report, using the validated Sleep Habits Survey for Adolescents^(^ ^48^ ^)^, with Actiheart data, as described previously^(^ ^47^ ^)^.

Family functioning was reported by participants using the twelve-item general functioning subscale of the McMaster Family Assessment Device^(^ ^49^ ^,^ ^50^ ^)^. Friendship quality was rated by participants using an eight-item questionnaire which assesses the availability, adequacy and intimacy of current friendships^(^ ^51^ ^)^. Self-esteem was measured using the Rosenberg self-esteem scale^(^ ^52^ ^,^ ^53^ ^)^.

Height (in metres), weight (in kilograms) and body-tissue impedance (in ohms; Tanita TBF-300 MA, Tokyo, Japan) were measured during school visits by trained research assistants following standard protocols. Fat-free mass, fat mass and body fat percentage were predicted based on impedance measurements using a pooled estimation approach, as described previously^(^ ^47^ ^)^. For descriptive purposes, overweight and obesity status were computed based on International Obesity Task Force BMI cut-offs^(^ ^54^ ^)^. Information regarding participants’ medication use was obtained from parents. Total energy intake was derived from the diet data, described above.

### Statistical analyses

All analyses were performed using the statistical software package Stata version 14. Student’s *t* tests and *χ* ^2^ tests were used to assess differences in sociodemographic and anthropometric variables between those included and excluded from the current analysis.

Multivariable linear regression models were used to test associations between dietary variables and depressive symptoms. Since adolescents were recruited through schools we tested whether school-level intraclass correlation coefficients might suggest use of multilevel analysis to adjust for clustering by school. However, intraclass correlation coefficients were low, which, together with a maximum cluster size of 50, suggests little impact of clustering on these analyses (intraclass correlation coefficient of 0·022 for baseline MFQ and <0·001 for follow-up MFQ)^(^ ^55^ ^)^; therefore adjustment for school-level clustering was not included.

In cross-sectional models, regression of the MFQ score at baseline *v*. (i) MDS, (ii) fruit and vegetable intake and (iii) fish intake was performed, adjusting for covariates. In prospective models, regression of the MFQ score at follow-up *v*. (i) MDS, (ii) fruit and vegetable intake and (iii) fish intake at baseline was performed, adjusting for MFQ score at baseline and covariates. Covariates were added to the models in stages, to understand the effects of covariate adjustment on model outputs. We explored the impact of order of addition of covariates. In the reported analyses, model 1 included only sociodemographic covariates, behavioural covariates were added in model 2, and psychosocial and anthropometric covariates were added in model 3, along with data on medication use and adjustment for total energy intake. Due to differences in mood (MFQ score) and diet quality (MDS) by sex, our final model (model 3) was also analysed stratified by sex.

Our primary analysis was a complete case analysis including those participants with data available on predictor and outcome variables and all covariates. We also conducted a secondary analysis of all participants for whom data on predictor and outcome variables were available (*n* 804), with imputation of missing covariate data (under the missing at random assumption), following recommendations from White *et al*.^(^ ^56^ ^)^.

## Results

### Descriptive characteristics

Of 926 participants who completed the dietary sub-study, 804 (87 %) had data on depressive symptoms at both baseline and follow-up. Further participants were dropped due to missing data on covariates (*n* 199) or misreporting of total energy intake (*n* 2), leaving 603 participants (65 % of dietary sub-study participants) contributing to these analyses. Those included in the analysis (*n* 603) did not differ from those excluded (*n* 323) by mean age (14·5 (sd 0·27) *v.* 14·5 (sd 0·28) years; *P*=0·54) or percentage body fat (21·7 *v.* 21·5 %; *P*=0·70). However, included participants were more likely to be female (included *v.* excluded: 60·0 *v.* 49·2 %; *P*=0·002) and from higher SES groups (included: 64·0 % high, 24·9 % medium, 11·1 % low SES; excluded: 58·1 % high, 22·7 % medium, 19·3 % low SES; *P*=0·003). Those included had a lower baseline MFQ score than those excluded (14·3 (sd 9·88) *v.* 16·2 (sd 10·2); *P*=0·008).

Table 1 presents descriptive characteristics of the study participants, as well as descriptive data on exposure and outcome variables. Both dietary variables and MFQ scores showed differences by sex. While total energy intake was higher among boys, the MDS was higher among girls, indicating better diet quality. MFQ scores were also higher among girls, indicating more depressive symptoms among girls than boys at baseline and follow-up.

**Table 1:** Characteristics of included study participants: adolescents recruited at age 14 years through secondary schools in Cambridgeshire and Suffolk, UK, from 2005 to 2007, and followed up at age 17 years (ROOTS study)

			Sex			
	All participants (*n* 603)		Male (*n* 241, 40·0 %)		Female (*n* 362, 60·0 %)	
	Mean	sd	Mean	sd	Mean	sd
Descriptive characteristics of the sample						
Age (years)	14·5	0·3	14·5	0·3	14·5	0·3
Socio-economic status (%)						
Low	11·1		13·3		9·7	
Medium	24·9		23·2		26·0	
High	64·0		63·5		64·4	
Weight status (%)						
Normal weight/underweight	81·8		82·6		81·2	
Overweight/obese	18·2		17·4		18·8	
Exposure and outcome variables						
Diet, baseline						
MDS	4·88	1·78	4·54	1·57	5·11***	1·87
Fruit and vegetable intake (servings/d)	3·34	2·51	3·43	2·69	3·28	2·39
Fish intake (servings/d)	0·12	0·20	0·14	0·23	0·10*	0·18
Energy intake (kJ/d)	7589·4	2128·8	8516·1	2185·3	6972·2	1851·4
Energy intake (kcal/d)	1813·9	508·8	2035·4	522·3	1666·4***	442·5
Depressive symptoms						
MFQ score, baseline	14·3	9·7	11·5	8·1	16·2***	10·2
MFQ score, 3-year follow-up	13·2	9·6	10·7	8·3	14·9***	10·0

MDS, Mediterranean diet score; MFQ, Moods and Feelings Questionnaire. Mean value was significantly different compared with males: *P<0·05, **P<0·01, ***P<0·001.

Table 2 presents further details on intake of dietary components of the MDS, by sex. Females had lower intakes of whole grains and red and processed meats compared with male participants.

**Table 2:** Intake of components of the Mediterranean diet, by sex, among adolescents recruited at age 14 years through secondary schools in Cambridgeshire and Suffolk, UK, from 2005 to 2007, and followed up at age 17 years (ROOTS study)

	All participants (*n* 603)		Male (*n* 241)		Female (*n* 362)		
	Median	IQR	Median	IQR	Median	IQR	*P* value†
Vegetable intake (g/d)	58·57	26·43–101·96	61·29	25·57–96·79	55·60	27·14–105·00	0·80
Legume intake (g/d)	0·00	0·00–19·29	0·00	0·00–19·29	0·00	0·00–13·21	0·93
Fruit intake (g/d)	155·00	57·14–280·48	165·00	53·33–293·29	152·64	58·57–272·86	0·36
Nut intake (g/d)	0·00	0·00–0·46	0·00	0·00–1·43	0·00	0·00–0·00	0·18
Whole grains intake (g/d)	14·29	0·00–45·00	17·14	0·00–53·57	11·43	0·00–42·50	**0**·**02**
Red and processed meat intake (g/d)	67·14	27·14–117·14	86·43	44·43–147·86	49·36	20·29–99·75	**<0**·**001**
Fish intake (g/d)	0·00	0·00–24·29	0·00	0·00–28·43	0·00	0·00–21·67	**0**·**04**
Ratio of monounsaturated to saturated fat	0·85	0·75–0·98	0·86	0·76–0·98	0·84	0·75–0·98	0·67
Alcohol (% consuming)	86·24	–	86·72	–	85·91	–	0·78

IQR, interquartile range.

† *P* value for the difference between males and females; statistically significant values (*P*<0·05) indicated in bold font.

### Associations between diet and depressive symptoms

Cross-sectional and prospective associations between MDS, fruit and vegetable intake and fish intake and depressive symptoms are shown in Table 3. There were no significant associations between diet quality (MDS), fruit and vegetable intake or fish intake and depressive symptoms at baseline, nor between baseline MDS, fruit and vegetable intake or fish intake and depressive symptoms at 3-year follow-up, after controlling for covariates. *R* ^2^ values indicated that for each explanatory variable, the final model (model 3) explained about 61 % of the variance in cross-sectional models and 26 % of the variance in prospective models.

**Table 3:** Cross-sectional and prospective associations between dietary variables and depressive symptoms among adolescents recruited at age 14 years through secondary schools in Cambridgeshire and Suffolk, UK, from 2005 to 2007, and followed up at age 17 years (ROOTS study)

	Unadjusted		Model 1: adjusted for demographic covariates		Model 2: additionally adjusted for behavioural covariates		Model 3: additionally adjusted for all other potential covariates		Model 3 (male)		Model 3 (female)	
	*β*	95 % CI	*β*	95 % CI	*β*	95 % CI	*β*	95 % CI	*β*	95 % CI	*β*	95 % CI
Cross-sectional associations												
MDS	0·15	−0·28, 0·58	−0·03	−0·46, 0·40	−0·02	−0·44, 0·39	−0·03	−0·31, 0·26	0·00	−0·47, 0·48	−0·08	−0·45, 0·29
Fruit and vegetables (servings/d)	**−0**·**40**	**−0·71**, **−0·10**	**−0**·**35**	**−0·65**, **−0·05**	−0·22	−0·51, 0·08	−0·07	−0·28, 0·14	−0·19	−0·47, 0·10	0·03	−0·28, 0·33
Fish (servings/d)	−2·99	−6·85, 0·88	−2·12	−5·91, 1·68	−1·88	−5·54, 1·78	0·52	−2·03, 3·06	2·07	−1·21, 5·35	−1·94	−5·88, 2·00
Prospective associations												
MDS	0·35	−0·03, 0·73	0·34	−0·04, 0·72	0·34	−0·05, 0·72	0·35	−0·04, 0·74	0·07	−0·58, 0·69	0·45	−0·07, 0·96
Fruit and vegetables (servings/d)	0·11	−0·16, 0·38	0·17	−0·10, 0·45	0·16	−0·12, 0·43	0·14	−0·15, 0·43	0·06	−0·32, 0·44	0·21	−0·22, 0·64
Fish (servings/d)	1·84	−1·56, 5·25	2·57	−0·83, 5·96	2·47	−0·95, 5·88	2·34	−1·15, 5·83	−0·09	−4·44, 4·27	4·20	−1·32, 9·72

MDS, Mediterranean diet score. Cross-sectional models perform the regression of MFQ score at baseline *v*. diet variables at baseline. Prospective models perform the regression of MFQ score at follow-up *v*. diet variables at baseline, with MFQ score at baseline included in all prospective models as a covariate. Model 1 includes baseline covariates: sex and socio-economic status. Model 2 includes baseline covariates: sex and socio-economic status, plus smoking level, alcohol consumption, physical activity and sleep. Model 3 includes the same covariates as model 2, as well as friendship quality, self-esteem, family functioning, medication use, percentage body fat and total energy intake (except MDS associations). One serving of fruit and vegetables=80 g; one serving of fish=140 g. Statistically significant associations (*P*<0·05) indicated in bold font.

Cross-sectional associations between fruit and vegetable consumption and depressive symptoms were seen in the unadjusted and minimally adjusted model (model 1) but were attenuated to below significance following adjustment for behavioural covariates (model 2). In particular, smoking and alcohol consumption contributed to attenuation of the association seen, while adjustment for physical activity and sleep had little impact on the model coefficient. Alternative model build-ups showed that addition of baseline energy intake as a covariate to model 1 also reduced observed associations to levels below significance (*β*=−0·27; 95 % CI −0·59, 0·04). Secondary analyses on imputed data sets did not alter the overall findings (see online supplementary material, Table S1).

## Discussion

### Summary of main findings

We found no prospective associations between MDS, fruit and vegetable intake or fish intake at age 14 years and depressive symptoms at age 17 years, adjusted for baseline depressive symptoms. At age 14 years, a negative cross-sectional association between fruit and vegetable intake and depressive symptoms was seen, but this was attenuated following adjustment for behavioural covariates (smoking and alcohol intake) or energy intake. No cross-sectional associations were found between MDS or fish intake and depressive symptoms.

### Strengths and limitations

The present study represents the strongest analysis of prospective associations between adolescent diet and depression to date. Its strengths lie in the detailed nature of the data, including high-quality measures of exposure and outcome, the data on a wide range of covariates and the longitudinal follow-up, allowing testing of prospective associations. The available evidence suggests that multi-day diet diaries are one of the most robust methods of collecting dietary data from adolescents^(^ ^57^ ^)^. One disadvantage of this method is in collection of data on infrequently consumed items (e.g. fish, legumes), which may not be consumed within the collection period, leading to loss of precision in estimation of usual intake of these items. We do not expect this to lead to any bias in our analyses, since among the population some members may record more, and some less, of their usual weekly consumption during the measurement period. Our outcome variable, the MFQ score, is a well-established and validated method of assessing depressive symptoms in this age group^(^ ^21^ ^)^. As a self-report measure the MFQ is subject to reporting biases. However, validation against clinician-led diagnostic interviews has shown moderate to high criterion validity and favourable comparison with other self-report measures^(^ ^21^ ^)^. In the present study, use of the same instrument at baseline and follow-up will ameliorate the impact of any time-invariant biases on prospective associations. Study data on a wide range of behavioural and social covariates allowed us to adjust for many putative confounders.

The ROOTS study population was not designed to be representative, but rather to sample a broad range of adolescents from the counties of Cambridgeshire and Suffolk. Compared with national UK data, the ROOTS cohort includes a higher proportion of participants from higher SES categories^(^ ^19^ ^)^. As such, the results are not generalizable to a national population, instead reflecting properties of a more wealthy, healthy and female population of teenagers. The current analysis included 603 adolescents, a comparatively large sample in this research area, particularly given the high-quality data on diet and covariates. Although the null associations observed could be due to type II error, the narrow confidence intervals for associations of MDS and fruit and vegetable servings with MFQ indicate that this is unlikely in these analyses. Wider confidence intervals for fish consumption suggest that our findings here are less certain, and further research in larger samples may be warranted. A further limitation is that we can only study associations at times when diet and depressive symptoms were measured in this cohort. The MFQ asks about symptoms of depression over the past two weeks, so cannot capture changes in mood which may have occurred across a longer time frame.

### Comparison with previous evidence and implications of the findings

In the present study, using an MDS which incorporated adjustment for total energy intake, we found no associations between MDS and depressive symptoms. Previous evidence on associations between diet and depression in adolescence has been mixed. Two recent reviews support associations between healthy dietary patterns or higher quality diet and lower levels of depression in adolescence. However, many of the included studies show limitations in measures used and in adjustment for confounding^(^ ^14^ ^,^ ^15^ ^)^. Many previous studies assessing relationships between diet and depression have not used comprehensive measures of dietary intake, meaning that measures of diet quality could not incorporate adjustment for misreporting or adjust for confounding by reported energy intake, as recommended^(^ ^45^ ^)^. We suggest that previously reported associations may have been driven in part by the inability to adjust for energy intake and dietary misreporting; under-reporting of energy intake is higher in specific population groups, such as obese individuals, with some limited evidence for higher under-reporting among depressed individuals^(^ ^58^ ^)^.

In the current study, we did see a small negative cross-sectional association between intake of fruit and vegetables and depressive symptoms in the unadjusted model. This association persisted following adjustment for gender and SES, but was attenuated following adjustment for behavioural covariates, particularly smoking and level of alcohol consumption. Previous studies have shown associations of both smoking and alcohol consumption with depression in adolescents^(^ ^39^ ^,^ ^59^ ^–^ ^61^ ^)^ and there is substantial evidence reporting clustering of diet with other health-related behaviours^(^ ^40^ ^,^ ^41^ ^)^. Therefore, it is likely that these behaviours may act as confounders which drive observed associations between diet and depression. Other studies that have reported associations between fruit and vegetables or fish and depression have typically neglected to control for such behavioural covariates^(^ ^15^ ^)^.

We did not see any prospective associations between baseline MDS and MFQ at follow-up, after adjusting for baseline MFQ, suggesting that diet quality at age 14 years does not influence development of depressive symptoms between 14 and 17 years of age. To our knowledge, only three previous studies have assessed prospective associations between measures of diet quality and depression in adolescence. Two studies found no prospective associations between healthy diet scores with depression after adjustment for confounders^(^ ^17^ ^,^ ^18^ ^)^, while one study found prospective associations of healthy and unhealthy diet scores with a measure of depressive symptoms, but after adjustment for a more limited range of confounders^(^ ^16^ ^)^. Again, it may be that residual confounding by behavioural covariates is implicated in the reported associations.

Intakes of fruit and vegetables and fish among our sample were on average below those recommended for a healthy diet. The WHO dietary guidelines recommend consumption of at least five portions of fruit and vegetables daily^(^ ^62^ ^)^, while UK guidelines additionally recommend consumption of at least two portions of fish per week^(^ ^32^ ^)^. The median consumption in our sample was 59 g (0·7 servings) of vegetables, 155 g (1·9 servings) of fruit and 0 g (0 servings) of fish daily, with those at the 75th percentiles of consumption also below stated recommendations, suggesting that the majority of this population does not consume a diet in line with healthy guidelines.

Despite finding no prospective associations in the present study, we cannot say conclusively that there are no associations between diet quality and prospective development of depressive symptomology among adolescents. Our findings here are constrained by the age range of this cohort; a greater predictive effect of diet might be seen at younger ages or with a shorter or longer follow-up time than 3 years. Alternatively, it may be that diet is part of a ‘chains of risk’ model^(^ ^63^ ^)^ but does not itself directly induce depressive symptomology. Finally, in the current study we have looked at population-level associations; however, diet may show stronger associations in vulnerable population subgroups. Further longitudinal studies are needed to investigate associations between diet and depression in adolescents over different time periods, as well as how multiple health behaviours may interact and influence the development of depressive symptomology.

## Conclusions

Diet quality, intake of fruit and vegetables and intake of fish were not associated with depressive symptoms in our population, after adjustment for covariates. These findings suggest that during adolescence, a better diet quality may not contribute to reduced risk of current or future depressive symptoms. Further longitudinal studies are needed which investigate associations between adolescent diet and depression across different time frames and populations, always ensuring appropriate adjustment for confounders.
