# Supplementary material for: Diet quality and depressive symptoms in adolescence: no cross-sectional or prospective associations following adjustment for covariates
Source: Public Health Nutr. 2018 May 16;21(13):2376–84. doi: 10.1017/S1368980018001179 (PMC6137369; doi:10.1017/S1368980018001179)
Supplement: Supplementary file 1 [file S1368980018001179sup001.docx]

**Table S1**: Comparison of associations using complete case analysis of 603 participants, with analysis of 801 participants with complete exposure and outcome data and multiple imputation of covariates.

|  | | Model 3, complete case analysis | | Model 3, imputed covariates | |
| --- | --- | --- | --- | --- | --- |
|  |  | beta coefficient | 95% CIs | beta coefficient | 95% CIs |
| Cross-sectional | | | | | |
|  | MDS | -0.03 | -0.31,0.26 | 0.01 | -0.24, 0.27 |
|  | Fruit and veg servs/day | -0.07 | -0.28,0.14 | -0.07 | -0.26, 0.11 |
|  | Fish servs/day | 0.52 | -2.03,3.06 | 0.01 | -2.34, 2.36 |
| Prospective | | | | | |
|  | MDS | 0.35 | -0.04, 0.74 | 0.15 | -0.20, 0.50 |
|  | Fruit and veg servs/day | 0.14 | -0.15,0.43 | 0.09 | -0.17, 0.35 |
|  | Fish servs/day | 2.34 | -1.15,5.83 | 2.77 | -0.47, 6.00 |

Footnote:

Abbreviations: MDS, Mediterranean Diet Score; servs/day, servings per day.

Cross-sectional models regress MFQ at baseline on diet variables at baseline. Prospective models regress MFQ at follow-up on diet variables at baseline, with MFQ at baseline included in all prospective models as a covariate.

Model 3 includes baseline covariates: sex, SES, smoking level, alcohol consumption, physical activity, sleep, friendship quality, self-esteem, family functioning, medication use, % body fat, total energy intake (except MDS associations).

80g F&V serving, 140g Fish serving
